# Effect of UV-C Irradiation on the Shelf Life of Fresh-Cut Potato and Its Sensory Properties after Cooking

**DOI:** 10.17113/ftb.60.02.22.7182

**Published:** 2022-06

**Authors:** Zdenka Pelaić, Zrinka Čošić, Maja Repajić, Sandra Pedisić, Zoran Zorić, Mario Ščetar, Kata Galić, Branka Levaj

**Affiliations:** 1 Faculty of Food Technology and Biotechnology, University of Zagreb, Petra Kasandrića 3, 23000 Zadar, Croatia; 2 Faculty of Food Technology and Biotechnology, University of Zagreb, Pierottijeva 6, 10000 Zagreb, Croatia

**Keywords:** potato cv. Birgit, firmness, CIELAB colour, sodium ascorbate treatment, vacuum packaging, principal component analysis (PCA)

## Abstract

**Research background:**

Potato tissue is damaged during fresh-cut production, which makes fresh-cut potato susceptible to the quality loss and microbiological spoilage. At the same time, such products are desirable due to their convenience; however, they are extremely sensitive and have short shelf life. The main challenge of the fresh-cut potato industry is to find possibilities to overcome these drawbacks. UV-C treatment, known for its antibacterial activity, is a promising technique and it shows a potential to improve shelf life of fresh-cut potato products.

**Experimental approach:**

The influence of the UV-C treatment on the safety and quality, as well as sensory traits of fresh-cut potato (*Solanum tuberosum* L. cv. Birgit) during storage was examined. For this purpose, 0-, 3-, 5- and 10-min UV-C irradiation was applied on vacuum-packed potato slices pretreated with sodium ascorbate solution. During 23 days of storage at (6±1) °C, microbiological, physicochemical and sensory properties of raw samples were monitored, along with sensory properties of boiled and fried fresh-cut potatoes.

**Results and conclusions:**

The 5- and 10-min UV-C treatments significantly reduced microbial growth, increased total solids and lightness (L*), and positively affected odour and firmness of raw potatoes. Cooked UV-C-treated samples were described with more pronounced characteristic potato odour and taste. Overall, UV-C-treated fresh-cut potato retained its good quality and sensory traits up to 15 days at (6±1) °C.

**Novelty and scientific contribution:**

To the best of our knowledge, this is the first scientific article dealing with the effect of UV-C light on durability (safety, quality and sensory traits) of fresh-cut potato cv. Birgit and its suitability for boiling and frying. In general, UV-C treatment is a known antimicrobial technique, but its application on fresh-cut potato is poorly explored. Results confirmed that vacuum-packed fresh-cut potato treated only with UV-C and sodium ascorbate as anti-browning agent, without the addition of chemical preservatives, had twofold longer shelf-life at (6±1) °C than the fresh-cut potato not treated with UV-C. Fresh-cut potato treated with UV-C retained good overall quality and sensory properties either raw, boiled or fried. Results of this study could also be useful for producers in terms of potential UV-C application as a strategy for prolonging the shelf-life of fresh-cut potato.

## INTRODUCTION

The popularity and commercial importance of the fresh-cut products are growing due to extreme convenience for the preparation of home meals, catering industry and in many other food services. The processing of fresh-cut fruits and vegetables includes only washing, trimming, peeling and/or cutting and packing to maintain their freshness and high nutritional value (*1*). During that process they are susceptible to microbial growth, water loss, off-odour, tissue softening, browning and general loss of quality, which makes them very perishable and limits their shelf life (*2*). During processing of fresh-cut products, enzymes and their substrates are delocalized due to cell integrity damage, which results in higher enzymatic activity responsible for oxidative reactions. These reactions lead to the formation of brown melanoid pigments (*3*).

Fresh-cut potato is a potentially interesting potato product (*4*) and many studies are focused on finding solutions to preserve the quality and safety of fresh-cut potato and to extend its shelf life. For this purpose, appropriate cultivar, antimicrobial and antibrowning agents, packaging materials and conditions as well as storage conditions have been investigated (*5*, *6*). According to our latest published study, fresh-cut potato cv. Birgit pretreated with sodium ascorbate solution and vacuum-packed showed promising results during 8 days of storage at 10 °C (*5*). Besides the above-mentioned approach, non-thermal UV-C technology has been investigated, especially in terms of prolonging shelf life by preventing microbial growth and enzyme activity (*7*). Antimicrobial effect of UV-C has a maximum effect at 254 nm and its effectiveness is based on structural changes in the DNA of microorganisms, caused by cross-linking between pyrimidine bases, which consequently contributes to the inability of transcription and replication of the cells (*8*). However, the irradiated plant tissue can be damaged using high UV-C doses (*9*). Besides, effectiveness of UV-C irradiation against enzyme activity depends on the applied dose and the sensitivity of enzymatic proteins, which is highly correlated with their nature (*10*, *11*). By exposing the enzyme to irradiation, their spatial structure can change, enabling better exposure of active sites, which leads to an initial increase in the enzyme activity (*12*). Thus, to extend the shelf life of fresh-cut products, it is necessary to evaluate the optimal doses of UV-C irradiation considering plant properties and the already mentioned antibrowning agents, packaging materials and packaging conditions, as well as storage conditions.

According to Teoh *et al*. (*6*), the optimal UV-C dose was 684 mJ/cm^2^ for potato slices dipped in ascorbic acid and calcium chloride solution, closed in permeable plastic boxes and stored for 10 days at 4 °C. This dose decreased the activity of polyphenol oxidase, phenylalanine ammonia lyase and peroxidase. Moreover, a significant decrease in browning and enzyme activity as well as increase in firmness were observed in the study of Xie *et al*. (*13*), where potato slices were treated with sodium acid sulphate, irradiated with UV-C for 3 min and stored in polyethylene bags for 25 days at 4 °C.

Selection of the packaging material is also very important, particularly if slices are packed and then UV-C treated. The permeability of materials to UV-C irradiation depends on the type of the used polymers as well as on its thickness. It was found that 40 µm thick polyamide/polyethylene laminate was permeable for 80% UV-C irradiation (*11*).

Furthermore, UV-C treatment showed a positive effect on soft rot prevention in potato seed tubers (*14*). Also, irradiation of tubers reduced the accumulation of fructose and glucose during cold storage, which consequently reduced the formation of toxic acrylamide during frying (*15*) and increased brightness of the fries (*16*).

However, although there is a number of studies that have dealt with the quality properties of cooked potatoes without UV-C treatment (*17*, *18*) or with UV-C pretreatment of tubers (*16*, *19*-*21*), reports regarding the effect of UV-C light on the quality and sensory attributes of raw and cooked fresh-cut potatoes are scarce.

Therefore, the aim of this study is to investigate the effect of different UV-C irradiation doses and 23 days of storage at (6±1) °C on microbial growth, quality and sensory properties of fresh-cut potato cv. Birgit, pretreated with sodium ascorbate solution and vacuum packed, as well as on the sensory properties of fresh-cut potatoes after boiling and frying.

## MATERIALS AND METHODS

### Plant material

Potato (*Solanum tuberosum* L.) tubers of cv. Birgit were harvested in Slavonia region, Croatia (45°40′N, 17°1′E) during 2019, treated with anti-sprouting agent (Gro Stop Basis and Gro Stop Fog, Certis Europe, Great Abington, UK) and stored for one month in the dark (8 °C and relative humidity approx. 100%) before analysis.

### Sample preparation

Undamaged and uniform potato tubers were selected, washed, drained, hand-peeled and sliced (0.4 cm) using a commercial slicer (SFS 1001 GR; Sencor, Říčany, Czech Republic). Immediately after slicing, potatoes were dipped in sodium ascorbate solution (2%, *m/V*) for 3 min according to the procedure described by Dite Hunjek *et al.* (*18*). After draining, potato samples (4-6 slices) were vacuum packed (SmartVac SV 750; Status, Metlika, Slovenia) in a single layer in the polyamide/polyethylene (PA/PE) double-layered (100 and 130 µm) vacuum pouches (Status).

### UV-C treatment

The potato slices were treated in an UV-C chamber (UVpro EKB 100; Orca GmbH, Kürten, Germany) equipped with 4 UV-C lamps (4×HNSL 24 W, maximal emission at 253.7 nm; UVpro). The samples were irradiated for 0 (control), 3 (3-UV-C), 5 (5-UV-C) and 10 min (10-UV-C) to obtain doses of 0, 162, 270 and 540 mJ/cm^2^ outside and 0, 108, 180 and 360 mJ/cm^2^ inside the vacuum bags (UVC *pro* radiometer; Orca GmbH). Afterwards, the untreated and UV-C-treated samples were stored at (6±1) °C and analysed at the beginning of the storage (day 0), on the 8th day, because in our previous study (*5*) we found that 8-day stability can be achieved using vacuum packing and sodium ascorbate treatment (under the same conditions as described in the paragraph *Sample preparation*), and on the 11th, 15th and 23rd days of storage. Experiment was done in duplicate.

### Determination of oxygen permeability of packaging

Oxygen permeability (cm^3^/(m^2^·day·kPa)) of packing was determined using manometric method on a permeability tester (GDP-C; Brugger Feinmechanik GmbH, Munich, Germany). The increase in the pressure during the test period was evaluated and displayed by an external computer. Data were recorded and permeability was calculated automatically. The sample temperature (23±1) °C was adjusted using an external thermostat (Haake F3 K circulating water bath chiller/heater; Haake GmbH, Karlsruhe, Germany). All measurements were carried out in duplicates.

### Microbiological analysis

Total aerobic mesophilic bacteria count (TAMBC) was determined at 30 °C according to HRN EN ISO 4833-1:2013 method (*22*). Dilutions were made with peptone water (0.1%, *m/V*) and surface plated (1 mL) in duplicate on a plate count agar (Biolife, Milan, Italy). The plates were incubated at (30±1) °C for (72±3) h in dry heat oven (FN-500; Nüve, Ankara, Turkey). Analyses were performed on raw samples and the results were expressed as mean value of log CFU/g.

### Determination of total solids, soluble solids and pH

The raw potato slices were homogenized (MSM89160 blender; Robert Bosch GmbH, Gerlingen-Schillerhöhe, Germany) and used for determination of total solids, soluble solids and acidity. Total solids were calculated as a percentage of the mass ratio before and after drying potato samples at (105±1) °C (FN-500; Nüve) to a constant mass, while soluble solids were determined by a digital refractometer (DR201-95; A. Krüss Optronic GmbH, Hamburg, Germany) at 20 °C and expressed as °Brix (g/100 g). The pH was measured by a pH meter (WTW Lab pH meter inoLab® pH 7110; Xylem Analytics Germany GmbH, Weilheim, Germany). All measurements were carried out in duplicates and results were expressed as mean value±standard error (S.E.).

### Firmness analysis

The firmness of raw fresh-cut potato samples was determined using a texture analyser (Fruit Texture Analyzer, Agrosta, Serqueux, France) with 5 kg load cell and 2 mm punch probe. High and low speeds were set to 1 mm/s and stroke after contact to 2 mm. Firmness was determined by measuring the maximum force (N) required to puncture the slices. The measurements were performed on two slices of each sample with 2 punctures on each slice and the results were expressed as mean value±S.E.

### Colour analysis

The colour of raw fresh-cut potato slices was measured by a colorimeter (CR-5; Konica Minolta, Tokyo, Japan), equipped with D65 light source and 2° standard observers using CIELAB colour parameters: L* (lightness), a* (red/green) and b* (yellow/blue). Measurements were performed on two slices of each sample and results were expressed as the mean value±S.E.

### Cooking treatments

Immediately after the treatment and on the 8th, 11th, 15th and 23rd day of storage, raw samples were cooked according to Dite Hunjek *et al.* (*18*). Samples were boiled in distilled water *Φ*(water, sample)=5:1 at 100 °C for 15 min. Other samples were fried in sunflower oil (*m*(sample)/*V*(oil))=120 g/L at initial temperature of 180 °C for 5 min. The surface moisture and oil of cooked potatoes were removed with paper towel.

### Sensory monitoring

Quantitative descriptive analysis (QDA) of raw, boiled and fried potato samples was conducted in a sensory laboratory equipped according to the ISO 8589:2007 (*23*) guidelines at ambient temperature (20 °C) by a panel of six trained people from the faculty and according to the ISO procedures 6658:2017 and 8586:2012 (*24*, *25*). Panellists had 3-day training before the evaluation in order to get acquainted with the product sensory descriptors and its evaluation. The panellists judged the quality and ranked each sample served at ambient temperature on coded plastic plates using a standard five-point scale from 1 (the lowest grade) to 5 (the highest grade) as described by Dite Hunjek *et al.* (*5*, *18*). Briefly, colour, as the browning intensity, was scored as follows: 1=no browning (white or cream), 2=no browning (yellow), 3=light browning, 4=average browning and 5=complete browning. Intensity of odour and off-odour was described as follows: 1=absent to 5=very pronounced, moistness from 1=very dry to 5=very moist and firmness from 1=very soft to 5=very firm. Additional sensory attributes of boiled and fried potatoes were evaluated: potato-, sweet, sour, salty, bitter and off-taste from 1=absent to 5=very pronounced. Creaminess of boiled potato was scored from 1=absence of creamy texture to 5=melting in the mouth, while oiliness and crispness, as fried potato attributes, were graded with 1=absent to 5=very pronounced. All tested attributes are given in the tables as mean value±S.E. (*N*=6).

### Statistical analysis

The statistical analysis by parametric statistical tests was carried out to observe the effect of the UV-C treatment and storage time on the quality properties of raw, boiled and fried potato. The TAMBC, soluble solids, total solids, pH, firmness, colour parameters and sensory attributes were dependent measurable variables, while UV-C treatment and storage time were independent variables. Dependent variables were analysed by multivariate analysis of variance (MANOVA), while differences between specific group means (equal sample sizes) were determined by applying Tukey’s HSD test. The analysis was performed using Statistica v. 8.0 software (*26*). In order to examine possible grouping of the samples, principal component analysis (PCA) was performed on the correlation matrix using XLSTAT v. 5.1 software (*27*), wherein principal components (PC) with eigenvalue >1 and variables with communalities ≥0.5 were considered. The significance level for all tests was α≤0.05.

## RESULTS AND DISCUSSION

### Influence of UV-C treatment on permeability of packing material

Although a slight increase of permeability of packing material (1200 and 1300 cm^3^/(m^2^·day·kPa)) was noticed for the samples 5-UV-C and 10-UV-C, respectively, this was not significantly different from control (900 cm^3^/(m^2^·day·kPa); data not shown). Sample 3-UV-C had identical value as the control. Tarek *et al.* (*28*) also concluded that the applied UV-C doses of 46.7–746 mJ/cm (for 0.5 to 8 min at 23 °C) did not affect surface properties of polyethylene (PE) film used for cucumber packing. It was also found that UV-C transmittance through polymeric films depends on their characteristics (such as thickness, composition, level of crystallinity and number of layers in the film). Thus, for example PE film (24.7 μm) shows transmittance of 75.5%, multilayer films composed of six or more layers exhibit 0% transmission (*29*), while PA/PE laminate is 80% permeable to UV-C (*11*), similar to polypropylene film (*30*). Although the effect of UV-C treatment on polymeric films has been investigated by several authors (*30*, *31*), it seems that this treatment does not affect barrier properties (*28*), while different observations were noticed for the mechanical and surface morphology of the polymers (*29*, *31*).

### Aerobic mesophilic bacterial count affected by UV-C treatment and storage time

**Fig. 1:**
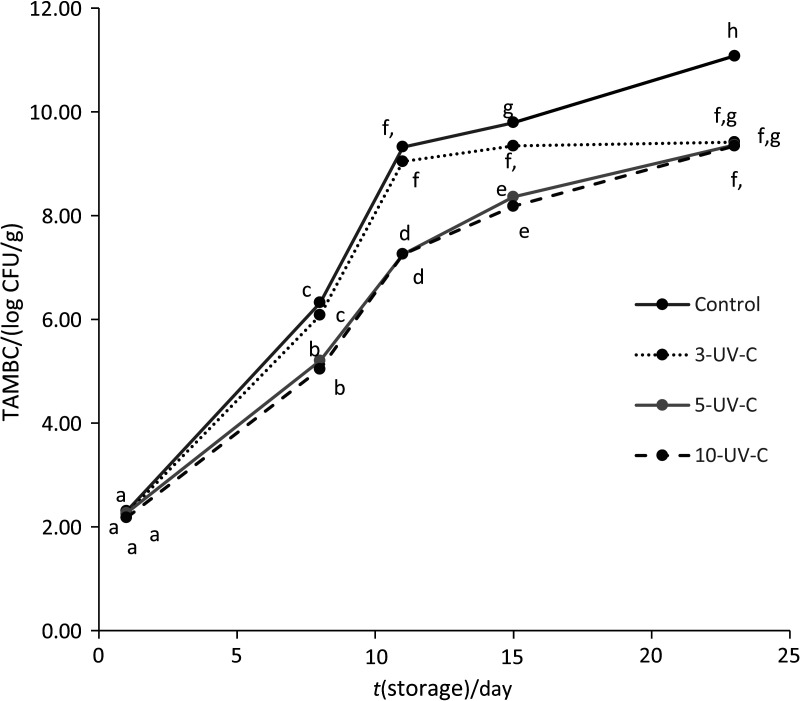
Total aerobic mesophilic bacteria count (TAMBC) of untreated and UV-C-treated raw fresh-cut potatoes during storage, expressed as mean values of log CFU/g (p<0.01, α≤0.05). 3-, 5- and 10-UV-C=samples treated with UV-C for 3, 5 and 10 min respectively

The TAMBC in untreated and UV-C-treated raw fresh-cut potato during storage is presented in Fig. 1. Statistical results showed significant differences (p<0.01) in TAMBC among fresh-cut potato samples. The initial microbial load of control sample was 2.30 log CFU/g. At the beginning of the storage, the lowest TAMBC was noticed in 10-UV-C samples (2.18 log CFU/g). When comparing all UV-C treatments with control throughout the storage period, the significant log CFU/g values decreased in 5- and 10-UV-C samples, especially until the 15th day. On that day, measured values for 5- and 10-UV-C samples were 8.36 and 8.17 log CFU/g, respectively. These results indicated that UV-C treatment longer than 5 min did not significantly improve the decontamination effect. Similar results were reported in a study of Manzocco *et al.* (*32*) on fresh-cut melon cubes. The possible reason could be low UV-C light transmittance through the tissue as well as the rough surface of the fresh-cut product, which can partially overshadow the microorganisms and thus reduce the effect of radiation (*32*, *33*). At the end of storage, all applied UV-C treatments were equally effective on TAMBC reduction in fresh-cut potatoes compared to the control. However, it should be mentioned that for this type of foodstuff (fresh-cut potato intended for further cooking) there is no information provided by the EC regulations (*34*, *35*) related to microbiological criteria regarding TAMBC. Similarly, the Croatian Agency for Agriculture and Food (*36*) issued borderline level of TAMBC only for ready-to-eat vacuum packed and refrigerated vegetables, and it is ≥10^8^ CFU/g.

### Effect of UV-C treatment and storage time on total and soluble solids, and pH of fresh-cut potato

As presented in Table 1, total solid content was affected by UV-C treatment (p=0.046), while storage time did not have a significant influence (p=0.054). Mean value of total solids in control sample was 21.7%. The highest values were obtained in 10–UV-C samples (23.2%) and generally on the 11th day of storage (23.1%). With regard to the UV-C treatment, all treated samples had higher total solid content, which increased with the increase of UV-C dose. The grand mean value of total solids was 22.24%, which was quite similar to that already reported (20.72%) by Dite Hunjek *et al*. (*5*) for cv. Birgit potatoes (harvested in 2018). The slight differences could be a result of different treatment, as well as crop year or growing conditions (*37*). Total solid content obtained in the present study represents an acceptable value in terms of frying, considering that potato dry matter content of 20-24% is appropriate for chips (*38*). Higher potato dry matter will result in harder crust and drier potato inside texture (*39*).

**Table 1:** The influence of UV-C treatment and storage time on total soluble (TSS) and total solids, pH, firmness and colour parameters of raw fresh-cut potatoes

Source of variation	*w*(total solid)/%	TSS/(g/100 g)	pH	Firmness/N	L*	a*	b*
Treatment	p=0.05*	p=0.01*	p<0.01*	p<0.01*	p<0.01*	p=0.22	p=0.33
Control	(21.7±0.4)^a^	(4.59±0.06)^b^	(5.64±0.01)^c^	(7.77±0.09)^c^	(71.8±0.4)^ab^	(1.6±0.2)^a^	(40.0±0.9)^a^
3-UV-C	(21.9±0.4)^ab^	(4.58±0.06)^ab^	(5.63±0.01)^bc^	(7.00±0.09)^a^	(71.1±0.4)^a^	(1.4±0.2)^a^	(38.4±0.9)^a^
5-UV-C	(22.2±0.4)^ab^	(4.44±0.06)^a^	(5.59±0.01)^ab^	(7.35±0.09)^ab^	(73.2±0.4)^b^	2.0±0.2)^a^	(40.3±0.9)^a^
10-UV-C	(23.2±0.4)^c^	(4.30±0.06)^a^	(5.57±0.01)^a^	(7.37±0.09)^b^	(73.0±0.4)^b^	(2.0±0.2)^a^	(40.5±0.9)^a^
*t*(storage)/day	p=0.05	p<0.01*	p<0.01*	p=0.14	p=0.38	p=0.10	p=0.25
0	(21.9±0.1)^a^	(4.80±0.07)^b^	(5.99±0.01)^d^	(7.4±0.1)^a^	(71.6±0.5)^a^	(1.7±0.3)^a^	(4077±1.0)^a^
8	(21.8±0.4)^a^	(4.40±0.07)^a^	(5.62±0.01)^c^	(7.5±0.1)^a^	(72.8±0.5)^a^	(2.2±0.3)^a^	(39.9±1.00)^a^
11	(23.1±0.4)^a^	(4.26±0.07)^a^	(5.51±0.01)^b^	(7.4±0.1)^a^	(71.9±058)^a^	(1.3±0.3)^a^	(39.6±1.0)^a^
15	(21.6±0.4)^a^	(4.18±0.07)^a^	(5.42±0.01)^a^	(7.2±0.1)^a^	(72.6±0.5)^a^	(219±0.3)^a^	(41.0±1.0)^a^
23	(22.9±0.4)^a^	(4.75±0.07)^b^	(5.50±0.01)^b^	(7.3±0.1)^a^	(72.4±0.5)^a^	(1.5±0.3)^a^	(37.9±1.0)^a^
Grand mean	22.24	4.48	5.61	7.37	72.24	1.74	39.80

*Statistically significant variable at α≤0.05. Results are expressed as mean value±S.E. Different letters in the same column mean statistically different values at α≤0.05. 3-UV-C, 5-UV-C and 10-UV-C=samples treated with UV-C for 3, 5 and 10 min respectively

However, the UV-C treatment and storage time had a significant influence (p≤0.01) on total soluble solid content, which varied from 4.18 to 4.80 g/100 g (°Bx) (Table 1). In comparison with control (4.59 g/100 g), the total soluble solid content decreased with the increase of UV-C dosage, where the lowest value was measured in 10-UV-C sample (4.30 g/100 g). These results are in accordance with the results of Islam *et al.* (*40*), who treated tomatoes with UV-C. This could be related to the impact of UV-C on conjugated structural bonds of some soluble solids, which leads to their degradation or alteration (*41*). In this study, a significant decrease in soluble solid content was observed after 8 days of storage (4.40 g/100 g), after which it remained stable until the end of storage, when it significantly increased. Kasim and Kasim (*42*) also reported oscillations of total soluble solids during storage depending on the applied dose of UV-C on fresh-cut melon cubes.

UV-C treatment and storage time significantly affected the pH of raw fresh-cut potatoes (p<0.01), which ranged from 5.42 to 5.99. When compared to the control (5.64), the lowest pH value was observed in 10-UV-C samples (5.57) and after 15 days of storage (5.42) (Table 1). The pH decreased with the increase of UV-C dosage, similarly to the results of Islam *et al*. (*40*), who also reported an increase of the titratable acidity of treated tomatoes with the increase of UV-C doses. Moreover, pH also decreased during storage probably due to the respiration rate increase and CO_2_ production, which is in accordance with the results of Dite Hunjek *et al.* (*5*) and Rocha *et al*. (*43*). Lower pH can contribute to lower enzyme activity and consequently to the reduced intensity of browning (*44*).

### Firmness of fresh-cut potatoes influenced by UV-C treatment and storage time

Firmness was significantly affected by the UV-C treatment (p<0.01) without significant effect of storage duration (p=0.14) (Table 1). The firmness grand mean value was 7.37 N, which is in accordance with the results for cv. Birgit (7.42 N) (*18*). Control sample was described with the highest firmness value (7.77 N) as well as the samples on the 8th day of storage (7.5 N). The firmness of fresh-cut potatoes was lower in the UV-C-treated samples than of the control. However, increase of the UV-C dose caused firmness increase, which could be linked to the possible reduction of activity of plant cell wall degrading enzymes (*45*). A similar observation was also previously reported when fresh-cut pineapples were treated with UV-C (*46*).

### Colour of fresh-cut potatoes influenced by UV-C treatment and storage time

The effect of UV-C treatment and storage time on the colour parameters of raw fresh-cut potatoes is shown in Table 1, where it can be observed that UV-C significantly affected only L* (p<0.01), while storage period had no significant effect on the colour during 23 days (p>0.05). The L* values were in the range from 71.1 to 73.2, a* values from 1.4 to 2.0 and b* values from 38.4 to 40.5 (Table 1). The lightness was considerably higher of 5-UV-C and 10-UV-C and lower of 3-UV-C samples than of control. Similar trend was also noticed in UV-C-treated watermelon, where L* values increased with the increase of the applied UV-C dose (*33*). This occurrence could be associated with the effect of UV-C light on the inactivation of enzymes such as polyphenol oxidase or with reduced carotenoid content (*47*). In this study the parameter b*, whose positive values describe yellow colour and usually reflect a presence of carotenoids in the potato (*48*), was not significantly reduced. The obtained colour parameters for fresh-cut potatoes are consistent with the European Cultivated Potato Database (*49*) data, where the colour of tuber flesh cv. Birgit is listed as yellow and also very resistant to enzymatic browning.

### Sensory attributes of raw, boiled and fried fresh-cut potato affected by UV-C treatment and storage time

#### Raw fresh-cut potato samples

All sensory attributes of raw fresh-cut potatoes were significantly affected by UV-C treatment and storage time (p<0.05), except moistness (p>0.05) (Table 2). The colour was rated from 1.58 in 3-UV-C to 1.98 in control, indicating negligible occurrence of browning. Furthermore, all UV-C-treated samples showed a significant discoloration and were graded as brighter than control, which is in accordance with previously discussed L* values (Table 1). Similar results were also reported by Manzocco *et al.* (*32*). The 10-UV-C samples showed more pronounced odour and less pronounced off-odour than other samples. All UV-C-treated samples were less firm than control, but the most pronounced reduction in firmness was observed for 3–UV-C samples, which is in accordance with the results measured by the instrument (Table 1).

**Table 2:** The influence of UV-C treatment and storage time on sensory properties of raw fresh-cut potatoes

Source of variation	Browning	Odour	Off-odour	Moistness	Firmness
Treatment	p<0.01*	p<0.01*	p<0.01*	p=0.09	p=0.03*
Control	(1.98±0.04)^b^	(3.40±0.03)^a^	(1.53±0.03)^b^	(1.55±0.02)^a^	(4.97±0.05)^b^
3-UV-C	(1.58±0.04)^a^	(3.47±0.03)^a^	(1.53±0.03)^b^	(1.52±0.02)^a^	(4.75±0.05)^a^
5-UV-C	(1.66±0.04)^a^	(3.48±0.03)^a^	(1.43±0.03)^b^	(1.50±0.02)^a^	(4.82±0.05)^ab^
10-UV-C	(1.65±0.04)^a^	(3.67±0.03)^b^	(1.17±0.03)^a^	(1.50±0.02)^a^	(4.85±0.05)^ab^
*t*(storage)/day	p<0.01*	p<0.01*	p<0.01*	p=0.06	p=0.01*
0	(1.50±0.05)^a^	(4.00±0.04)^b^	(1.00±0.03)^a^	(1.56±0.02)^a^	(4.65±0.06)^a^
8	(1.50±0.05)^a^	(4.00±0.04)^b^	(1.00±0.03)^a^	(1.52±0.02)^a^	(4.92±0.06)^b^
11	(1.73±0.05)^b^	(3.98±0.04)^b^	(1.00±0.03)^a^	(1.50±0.02)^a^	(4.90±0.06)^b^
15	(1.94±0.05)^c^	(3.98±0.04)^b^	(1.02±0.03)^a^	(1.50±0.02)^a^	(4.88±0.06)^b^
23	(1.94±0.05)^c^	(1.56±0.04)^a^	(3.06±0.03)^b^	(1.50±0.02)^a^	(4.90±0.06)^b^
Grand mean	1.72	3.51	1.42	1.52	4.85

*Statistically significant variable at α≤0.05. Results are expressed as mean±S.E. Different letters in the same column mean statistically different values at α≤0. 3-UV-C, 5-UV-C and 10-UV-C=samples treated with UV-C for 3, 5 and 10 min respectively

During storage, potato colour was scored from 1.50 to 1.94 indicating no degradation in terms of browning. The odour was stable until the 15th day of storage, but at the end of storage the development of off-odour was notable. The lowest firmness was observed at the beginning of storage, but by the end of storage the fresh-cut potatoes maintained uniform firmness. Generally, the results of this study showed that UV-C treatment preserved sensory attributes of colour, odour, moistness and firmness of raw fresh-cut potatoes during 15 days of storage. However due to the off-odour development, the samples were not sensorially acceptable at the end of storage.

#### Boiled fresh-cut potato samples

Table 3 shows that the majority of the evaluated sensory attributes of boiled potatoes were significantly affected by UV-C treatment and storage duration (p<0.05). Sour, bitter and off-taste were not influenced by storage time nor off-odour and moistness by UV-C treatment (p>0.05). As observed for raw samples, all UV-C-treated samples had brighter colour. The 5- and 10-UV-C samples had more intense boiled potato odour, sweet, salty and potato taste. The desirable boiled potato flavour is a result of many naturally present characteristic compounds (glutamic and other amino acids) and the ones produced during cooking (*e.g*. guanosine-5'-monophosphate and other 5'-ribonucleotides). Many other components such as methional, aliphatic alcohols and aldehydes also contribute to potato flavour. Besides, the desirable flavour of boiled potato derives from 2-isopropyl-3-methoxypyrazine, a compound with extremely low threshold present in raw and boiled potato (*50*-*52*). Obviously, UV-C treatment did not have a negative impact on flavour compounds, even stimulated their formation or better expression. All UV-C-treated samples had lower firmness and more pronounced creaminess than the control, where higher decrease in firmness and increase in creaminess were noticed when the UV-C dose was increased. The increased UV-C dose could probably induce some structural changes in the potato tissue, which can consequently be observed in a softer texture of the boiled potatoes. The softening degree of the boiled potatoes during cooking is influenced by starch characteristics such as amylose to amylopectin ratio, cell separation and cell wall softening (*37*). Some functional properties of starch can be changed as a result of prolonged UV-C treatment, such as capability of absorbing and holding water during gelatinization, reduction in amylose content, appearance of fractures and exocorrosion on the surface of the starch granule or a drop of crystallinity (*53*).

**Table 3:** The influence of UV-C treatment and storage time on sensory properties of boiled fresh-cut potatoes

Source of variation	Browning	Odour	Off-odour	Moistness	Firmness	Creaminess	Potato taste	Sweet taste	Sour taste	Salty taste	Bitter taste	Off-taste
Treatment	p<0.01*	p<0.01*	p=0.17	p=0.70	p<0.01*	p<0.01*	p<0.01*	p<0.01*	p=0.39	p<0.01*	p=0.53	p=0.57
Control	(2.40±0.05)^c^	(4.70±0.03)^a^	(1.07±0.03)^a^	(2.07±0.03)^a^	(2.30±0.08)^c^	(3.70±0.07)^a^	(4.35±0.05)^a^	(1.05±0.04)^a^	(1.05±0.03)^a^	(1.00±0.04)^a^	(1.03±0.02)^a^	(1.00±0.01)^a^
3-UV-C	(1.95±0.05)^a^	(4.78±0.03)^a^	(1.08±0.03)^a^	(2.08±0.03)^a^	(1.97±0.08)^b^	(4.23±0.07)^b^	(4.93±0.05)^b^	(1.12±0.04)^ab^	(1.00±0.03)^a^	(1.00±0.04)^a^	(1.00±0.02)^a^	(1.00±0.01)^a^
5-UV-C	(2.13±0.05)^b^	(4.92±0.03)^b^	(1.03±0.03)^a^	(2.12±0.03)^a^	(1.72±0.08)^b^	(4.38±0.07)^b^	(4.92±0.05)^b^	(1.23±0.04^b^	(1.03±0.03)^a^	(1.20±0.04)^b^	(1.00±0.02)^a^	(1.02±0.01)^a^
10-UV-C	(2.03±0.05)^ab^	(5.00±0.03)^b^	(1.00±0.03)^a^	(2.10±0.03)^a^	(1.12±0.08)^a^	(4.72±0.07)^c^	(4.92±0.05)^b^	(1.45±0.04)^c^	(1.00±0.03)^a^	(1.50±0.04)^b^	(1.02±0.02)^a^	(1.02±0.01)^a^
*t*(storage)/day	p<0.01*	p<0.01*	p<0.01*	p<0.01*	p<0.01*	p<0.01*	p=0.03*	p<0.01*	p=0.36	p<0.01*	p=0.13	p=0.10
0	(1.50±0.05)^a^	(5.00±0.04)^c^	(1.00±0.03)^a^	(2.00±0.03)^a^	(1.46±0.09)^a^	(4.58±0.07)^b^	(4.69±0.05)^a^	(1.10±0.05)^a^	(1.00±0.03)^a^	(1.08±0.05)^ab^	(1.00±0.02)^a^	(1.00±0.01)^a^
8	(1.92±0.05)^b^	(4.52±0.04)^a^	(1.00±0.03)^a^	(2.17±0.03)^b^	(2.10±0.09)^b^	(4.13±0.07)^a^	(4.83±0.05)^a^	(1.06±0.05)^a^	(1.00±0.03)^a^	(1.48±0.05)^c^	(1.00±0.02)^a^	(1.00±0.01)^a^
11	(1.67±0.05)^a^	(5.00±0.04)^c^	(1.00±0.03)^a^	(2.30±0.03)^b^	(1.83±0.09)^bc^	(4.23±0.07)^a^	(4.88±0.05)^a^	(1.75±0.05)^b^	(1.00±0.03)^a^	(1.27±0.05)^b^	(1.00±0.02)^a^	(1.00±0.01)^a^
15	(2.65±0.05)^c^	(5.00±0.04)^c^	(1.00±0.03)^a^	(2.00±0.03)^a^	(1.58±0.09)^ab^	(4.23±0.07)^a^	(4.85±0.05)^a^	(1.10±0.05)^a^	(1.04±0.03)^a^	(1.04±0.05)^a^	(1.00±0.02)^a^	(1.00±0.01)^a^
23	(2.92±0.05)^d^	(4.73±0.04)^b^	(1.23±0.03)^b^	(2.00±0.03)^a^	(1.90±0.09)^bc^	(4.13±0.07)^a^	(4.65±0.05)^a^	(1.04±0.05)^a^	(1.06±0.03)^a^	(1.00±0.05)^a^	(1.06±0.02)^a^	(1.04±0.01)^a^
Grand mean	2.13	4.85	1.05	2.09	1.78	4.26	4.78	1.21	1.02	1.18	1.01	1.01

*Statistically significant variable at α≤0.05. Results are expressed as mean±S.E. Different letters in the same column mean statistically different values at α≤0.05. 3-UV-C, 5-UV-c and 10-UV-C=samples treated with UV-C for 3, 5 and 10 min respectively

After the 15th day of storage browning was slightly more pronounced. Throughout the storage the odour was highly rated, while the off-odour was more pronounced only at the end of storage, and it received lower scores in boiled than in the raw samples. This could be explained by the volatility of compounds responsible for off-odour of raw potato. This was also observed previously by Dite Hunjek *et al.* (*5*). The firmness and creaminess showed variations in scores during storage; however, at the beginning of storage firmness was rated with the lowest scores and creaminess achieved the highest scores. On the 11th day, sweet taste was the most evident compared to other days, while a salty taste was the most prominent on the 8th day. Generally, boiled 5- and 10-UV-C samples were characterised by desirable odour, creaminess and taste, as well as appropriate colour and acceptable firmness. These favourable sensory attributes were preserved for 23 days of storage.

#### Fried fresh-cut potato samples

Most of the analysed sensory attributes of fried potato were significantly affected by the UV-C treatment and storage time (p<0.01), with an exception of off-odour, crispiness, sour, bitter and off-taste (Table 4). Oiliness was significantly influenced only by storage time (p<0.01), but numerical differences were very slight (in a range from 1.00 to 1.13). All UV-C-treated samples had slightly brighter colour (2.11 to 2.15) after frying than fried control samples (2.33), and browning was not observed. According to the results of Sobol *et al*. (*16*), UV-C irradiation applied on potato tubers increased the brightness of the fried potatoes, which is in line with present results. Lin *et al*. (*15*) reported lower content of fructose and glucose in irradiated tubers during storage. During processing at high temperatures, reducing sugars and amino acids participate in Maillard’s reactions, which are responsible for colour and volatile compound formation in fried products (*54*). Presumably, increased brightness could be linked to lowering of reducing sugars caused by UV-C treatment. Firmness of 10-UV-C samples significantly decreased, as it was observed in boiled ones (Table 3). Odour and potato, sweet and salty taste significantly increased in fried 10-UV-C potatoes. Potato taste intensity increased with applied UV-C dose, while potato off-taste was not pronounced, a similar observation was for boiled fresh-cut potatoes.

**Table 4:** The influence of UV-C treatment and storage days on sensory properties of fried fresh-cut potatoes

Source of variation	Browning	Odour	Off-odour	Oiliness	Firmness	Crispiness	Potato taste	Sweet taste	Sour taste	Salty taste	Bitter taste	Off-taste
Treatment	p<0.01*	p<0.01*	p=0.53	p=0.85	p<0.01*	p=0.53	p<0.01*	p<0.01*	p=0.53	p<0.01*	p=0.56	p=0.53
Control	(2.33±0.03)^b^	(4.63±0.04)^a^	(1.03±0.02)^a^	(1.07±0.03)^a^	(2.32±0.07)^b^	(2.00±0.02)^a^	(3.98±0.05)^a^	(1.00±0.04)^a^	(1.03±0.02)^a^	(1.00±0.04)^a^	(1.05±0.03)^a^	(1.03±0.02)^a^
3-UV-C	(2.11±0.03)^a^	(4.75±0.04)^ab^	(1.02±0.02)^a^	(1.03±0.03)^a^	(2.40±0.07)^b^	(2.02±0.02)^a^	(4.32±0.05)^b^	(1.00±0.04)^a^	(1.02±0.02)^a^	(1.00±0.04)^a^	(1.03±0.03)^a^	(1.02±0.02)^a^
5-UV-C	(2.15±0.03)^a^	(4.80±0.04)^bc^	(1.00±0.02)^a^	(1.05±0.03)^a^	(2.30±0.07)^ab^	(2.03±0.02)^a^	(4.33±0.05)^b^	(1.15±0.04)^b^	(1.00±0.02)^a^	(1.17±0.04)^b^	(1.00±0.03)^a^	(1.00±0.02)^a^
10-UV-C	(2.15±0.03)^a^	(4.93±0.04)^c^	(1.00±0.02)^a^	(1.05±0.03)^a^	(2.03±0.07)^a^	(2.00±0.02)^a^	(4.68±0.05)^c^	(1.52±0.04)^c^	(1.00±0.02)^a^	(1.40±0.04)^c^	(1.00±0.03)^a^	(1.00±0.02)^a^
*t*(storage)/ day	p<0.01*	p<0.01*	p=0.53	p<0.01*	p<0.01*	p=0.53	p<0.01*	p<0.01*	p=0.53	p<0.01*	p=0.55	p=0.53
0	(2.00±0.04)^a^	(5.00±0.05)^c^	(1.00±0.01)^a^	(1.00±0.04)^a^	(2.27±0.08)^ab^	(2.00±0.02)^a^	(4.21±0.05)^a^	(1.29±0.05)^b^	(1.00±0.02)^a^	(1.20±0.04)^bc^	(1.00±0.03)^a^	(1.00±0.02)^a^
8	(2.00±0.04)^a^	(4.58±0.05)^a^	(1.00±0.02)^a^	(1.00±0.04)^a^	(2.15±0.08)^ab^	(2.00±0.02)^a^	(4.54±0.05)^c^	(1.29±0.05)^b^	(1.00±0.02)^a^	(1.25±0.04)^c^	(1.00±0.03)^a^	(1.00±0.02)^a^
11	(2.00±0.04)^a^	(4.63±0.05)^a^	(1.00±0.02)^a^	(1.00±0.04)^a^	(2.46±0.08)^b^	(2.04±0.02)^a^	(4.17±0.05)^a^	(1.13±0.05)^ab^	(1.00±0.02)^a^	(1.03±0.04)^a^	(1.00±0.03)^a^	(1.00±0.02)^a^
15	(2.52±0.04)^b^	(4.75±0.05)^ab^	(1.02±0.02)^a^	(1.13±0.04)^b^	(2.35±0.08)^ab^	(2.02±0.02)^a^	(4.25±0.05)^ab^	(1.13±0.05)^ab^	(1.04±0.02)^a^	(1.08±0.04)^ab^	(1.04±0.03)^a^	(1.02±0.02)^a^
23	(2.42±0.04)^b^	(4.94±0.05)^bc^	(1.04±0.02)^a^	(1.13±0.04)^b^	(2.08±0.08)^a^	(2.00±0.02)^a^	(4.48±0.05)^bc^	(1.00±0.05)^a^	(1.02±0.02)^a^	(1.00±0.04)^a^	(1.06±0.03)^a^	(1.04±0.02)^a^
Grand mean	2.17	4.78	1.01	1.05	2.26	2.01	4.33	1.17	1.01	1.14	1.02	1.01

*Statistically significant variable at α≤0.05. Results are expressed as mean±S.E. Different letters in the same column mean statistically different values at α≤0.05. 3-UV-C, 5-UV-C and 10-UV-C=samples treated with UV-C for 3, 5 and 10 min respectively

Even though storage time showed a significant effect (p<0.01) on more than half of the evaluated properties, numerical differences were very slight. The browning scores were in the range of 2.00–2.52, and they were more pronounced on the 15th and 23rd days, like in boiled samples. Moreover, the sweet and salty taste of fried potatoes decreased and oiliness increased with storage time. The potato taste and odour were highly scored, and off-odour was not noticed regardless of the fresh-cut potato storage duration. These results indicate that the observed changes in off-odour of stored raw fresh-cut potatoes do not have an influence on the odour of fried potatoes, which is in accordance with observations for boiled potatoes. Generally, UV-C treatments positively affected the taste, odour and colour formation in fried potatoes regardless of storage time.

### Results of PCA analysis of the applied UV-C treatment and storage time

**Fig. 2:**
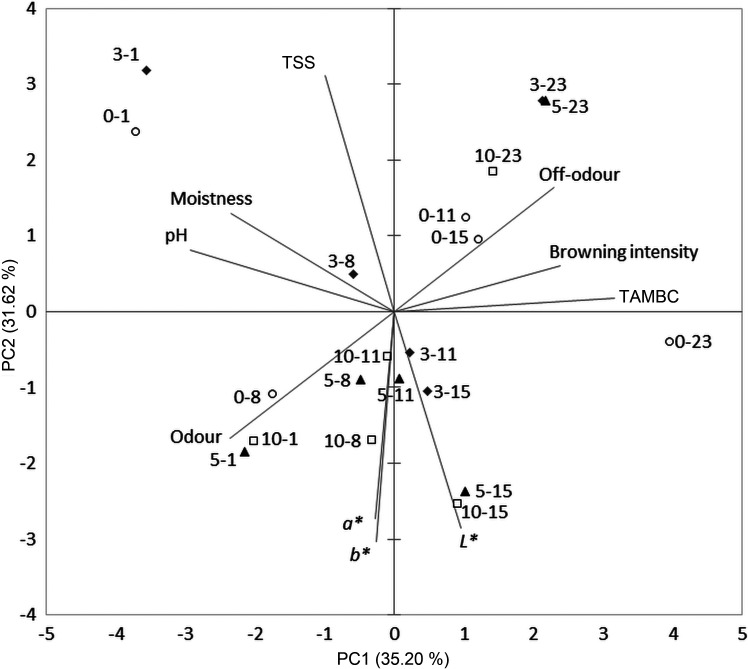
Biplot related to the raw fresh-cut potatoes. The first number in the sample label indicates UV-C treatment (min) and the second number indicates storage day. TAMBC=total aerobic mesophilic bacteria count, TSS=total soluble solids

**Fig. 3:**
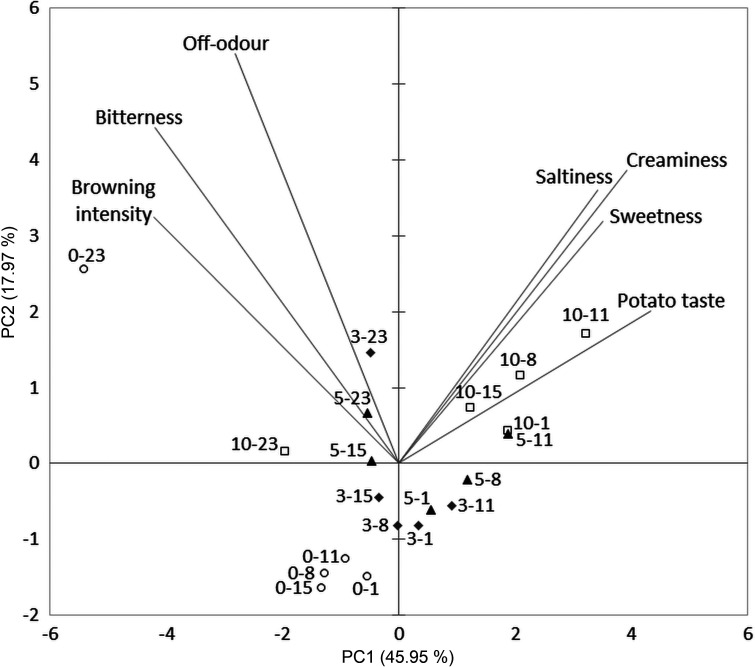
Biplot related to the boiled fresh-cut potatoes. The first number in the sample label indicates UV-C treatment (min) and the second number indicates storage day

**Fig. 4:**
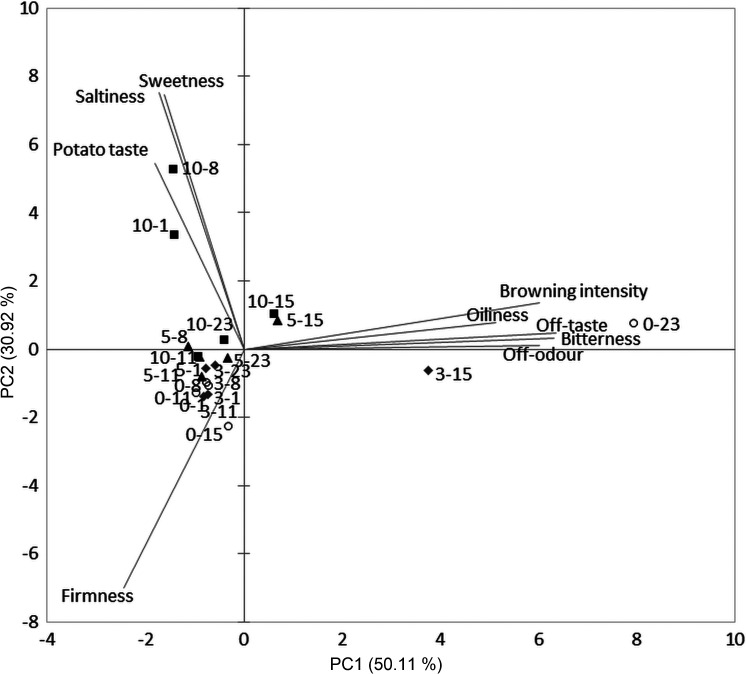
Biplot related to the fried fresh-cut potatoes. The first number in the sample label indicates UV-C treatment (min) and the second number indicates storage day

PCA was used to visualize relations among the analysed parameters and to determine possible grouping of raw, boiled and fried fresh-cut potato samples in relation to the applied UV-C treatment and storage time (Fig. 2, Fig. 3 and Fig. 4, respectively).

In terms of raw fresh-cut potato samples, TAMBC, pH, total soluble solids, L*, a*, b* and all sensory attributes except sensorial firmness were included in the test (Fig. 2). PC1 and PC2 together described 66.81% of the total data variance, where PC1 correlated very strongly with TAMBC (r=0.908), strongly with pH (r=-0.842) and moderately with browning intensity (r=0.686), odour (r=-0.678), off-odour (r=0.659) and moistness (r=-0.672). On the other hand, PC2 showed a very strong correlation with total soluble solids (r=0.848) and b* (r=-0.826) and strong correlation with L* (r=-0.778) and a* (r=-0.743), while moderate correlation was present between this PC and odour (r=-0.453) as well as off-odour (r=0.444). Considering UV-C treatment duration, almost all 5- and 10-UV-C-treated raw samples were placed among negative PC2 values since they received higher scores for colour parameters and odour. Moreover, 3-UV-C samples were not perceived as a separate group, while 0-UV-C samples were distributed mainly among positive PC1 and PC2 values. Furthermore, grouping was observed in relation to storage time, where all samples from the 23rd day of storage were also placed in the upper right quadrant, characterized by higher values of TAMBC, browning and off-odour. The 0-UV-C samples taken on the 11th and 15th days of storage were also positioned in this part of the factorial plane.

Considering the boiled fresh-cut potato samples, browning intensity, odour, moistness, sensorial firmness, creaminess, potato taste and bitterness were selected as PCA-active variables and PC1 and PC2 explained 63.92% of the total data variance (Fig. 3). PC1 showed a strong correlation with browning intensity (r=-0.750), creaminess (r=0.699), potato taste (r=0.770), sweetness (r=0.626), saltiness (r=0.611) and bitterness (r=-0.746) as well as a moderate correlation with off-odour (r=-0.501), while a strong/moderate correlation was present between PC2 and off-odour (r=0.602), creaminess (r=0.430), saltiness (0.402) and bitterness (r=0.493). Clear separation of the samples can be noticed with regard to UV-C treatment. The major distinction of the samples was observed for 10-UV-C-treated samples, which were distributed among the positive values of PC1 and PC2 and were characterized by positive sensorial attributes: creaminess, saltiness, sweetness and characteristic potato taste. On the other hand, almost all control samples were placed among the negative values of PC1 and PC2. Also, 3- and 5-UV-C samples were situated around the centre of the factorial plane. Besides, boiled fresh-cut potatoes on the 23rd storage day were again separated by negative PC1 values, and were correlated with scores for browning intensity, bitterness and off-odour, which were especially high in the control sample.

As for fried potato samples, browning intensity, off-odour, oiliness, sensorial firmness, potato taste, sweetness, saltiness, bitterness and off-taste were considered and the first two PCs described 81.03% of the total data variance (Fig. 4). A very strong/strong correlation was present between browning intensity (r=0.918), off-odour (r=0.918), bitterness (r=0.962), off-taste (r=0.969), oiliness (r=0.783) and PC1, while PC2 correlated strongly/very strongly with sensorial firmness (r=-0.838), sweetness (r=0.895), saltiness (r=0.901) and potato taste (r=0.654). The grouping of the fried potato samples in terms of UV-C treatment is rather poor, where only 10-UV-C samples, which received the highest scores for sweetness, saltiness and potato taste, were slightly distanced from the rest of the samples, especially from the samples fried at the beginning of the storage (1st and 8th day). Again, control sample from the 23rd day of storage was separated from the rest of the samples with the highest scores for undesirable sensory attributes, *i.e*. browning, oiliness, off-taste, bitterness and off-odour.

## CONCLUSIONS

UV-C technology is promising and it has a potential practical application in fresh-cut industry, especially since it has already been approved for application in food industry, specifically for liquid systems or surface disinfection. Furthermore, it is considered as environmentally friendly with low costs of energy, equipment and maintenance.

The results of this study could contribute to UV-C application in fresh-cut industry since UV-C treatment in combination with sodium ascorbate and vacuum packaging showed high efficiency in the reduction of microbial count in raw fresh-cut potato cv. Birgit during storage at (6±1) °C and in extension of its shelf life. UV-C treatments for 5 and 10 min were particularly effective. Generally, good quality and sensory attributes of fresh-cut potato were retained for up to 15 days of storage. The treatment also contributed to the reduction of browning and affected the odour of raw fresh-cut potatoes positively, and acceptable firmness was retained as well. Furthermore, UV-C-treated fresh-cut potatoes after boiling and frying were also sensorially desirable as they were characterized with more pronounced characteristic potato odour and taste than untreated samples.

On a potential large-scale production of fresh-cut potatoes UV-C treatment could present relatively short additional operation for ensuring safety and extended shelf life. Namely, it could be the final operation after potato slicing, treatment by antibrowning agents (*e.g.* sodium ascorbate solution) and after vacuum packaging. However, further investigation is needed in order to determine all parameters necessary to confirm the use of UV-C technology on a real scale in the fresh-cut potato industry.
